# Supplementary material for: Simulating COVID19 transmission from observed movement
Source: Sci Rep. 2022 Feb 23;12:3044. doi: 10.1038/s41598-022-07043-4 (PMC8866419; doi:10.1038/s41598-022-07043-4)
Supplement: Supplementary file 1 — Supplementary Information. [file 41598_2022_7043_MOESM1_ESM.pdf]

Supporting Material

1. DATA DESCRIPTION

Class characteristics are reported in Table S1.

Table S1. Statistical summary of the characteristics of different classes.

**Class**	Class A	Class B	Class C	Class D
**Number of observations**	13	12	5	4
**Average data collection length**	3.1 hours	3.2 hours	2.2 hours	2.3 hours
**Total number of children (girls)**	17 (8)	10 (4)	12 (5)	11 (7)
**Mean (SD) of number of present children**	12.83 (1.47)	8.50 (1.45)	10.6 (1.52)	8.25 (0.50)
**Range of number of present children**	11-15	7-10	9-12	8-9
**Total number of teachers**	3	2	3	3
**Mean (SD) of number of present teachers**	2.67 (0.49)	1.50 (0.52)	2.20 (0.45)	2.5 (0.58)
**Range of number of present teachers**	2-3	1-2	2-3	2-3
**Child Age (mean months)**	30	35	52	41

Note. Present refers to the number of individuals present on each day when data were collected.

2. CLASSROOM MEASUREMENTS

Classroom Density. The classroom density indexes the number of individuals in the classroom. As data are collected over multiple days in each classroom and the numbers of teachers and children present on each data vary, classroom density is computed, as a mean. The average number of people that appear in the classroom will be used. In addition, as the start and end time of the data collection could vary slightly by day, the mean number of individuals is weighted by the duration of the data collection periods. Thus, the classroom density can be formally defined as

$$\rho = \frac{\sum\limits_{d \in D} T_d \cdot N_d}{A \sum\limits_{d \in D} T_d}, \tag{S1}$$

where A is the area of the classroom, D is the set of data collection days, T_d is the duration of data collection period on d, and N_d is the number of individuals (both teachers and children) in the classroom on d. In addition, in Class A, the physical layout includes a terrace that individuals occupy during part of each data collection day. Thus, the computation of the classroom density involves splitting each day into two scenarios, namely "in classroom" and "in terrace," whose density is calculated separately. Formally, the classroom density of classroom A can be computed by

$$\rho = \frac{\sum\limits_{d \in D} \left(\frac{T_d^{Terrace}}{A_{Terrace}} + \frac{T_d^{Classroom}}{A_{Classroom}} \right) \cdot N_d}{\sum\limits_{d \in D} T_d^{Terrace} + T_d^{Classroom}}, \tag{S2}$$

where $T_d^{Terrace}$ and $T_d^{Classroom}$ the duration of individuals in the terrace and classroom, respectively, and $A_{Terrace}$ and $A_{Classroom}$ the area of the terrace and classroom, respectively. Since the transition time of people moving from the terrace to classroom is short, and vice versa, each data collection day is bisected into two scenarios by a simple condition whether more than half of the people are in the classroom or the terrace.

Transmission Rate for Each Observation. The simulation of COVID-19 transmission in the classroom begins with a patient zero who is infected outside of the classroom. Then, the likelihood of an individual i being infected in a classroom, is computed as the time average of the transmission rate between the person and all other infected persons in the classroom,

$$\hat{\beta}_i = \frac{1}{T_i} \sum_{t=1}^{T_i} \sum_{j \in I[t]} \beta(r_{ij}[t], \theta_i[t], \theta_j[t]), \tag{S3}$$

where the transmission rate of each pair $\beta(r_{ij}[t], \theta_i[t], \theta_j[t])$ is computed based on Eq. (1), the distance $r_{ij}[t]$ and orientation $\theta_i[t]$ and $\theta_j[t]$ are inferred from the observation data, T_i is the total time when the person i is present in the classroom and has not been infected, and $I[t]$ is the set of infected people in the classroom at time t. Then, for each simulation, the overall transmission rate is defined as the mean of average transmission rate of each person in the classroom, which can be formally defined as

$$\hat{\beta} = \frac{1}{|P|} \sum_{i \in P} \hat{\beta}_i, \tag{S4}$$

where P is the non-vaccinated individuals in the classroom, excepting patient zero. Since classroom durations vary slightly and individuals may be present in the classroom for varying periods, time is used to represent the transmission likelihood in a classroom based on the empirical data (as used in Figure 4). Therefore, we define the transmission likelihood $\hat{\beta}T$ of an observation as the mean of accumulated transmission rate of each person in the classroom, i.e.,

$$\hat{\beta}T = \frac{1}{|P|} \sum_{i \in P} \hat{\beta}_i T_i. \tag{S5}$$

3. CLASSROOM COVID-19 SIMULATION STATISTICS

A. Saturation and infection likelihood

Table S2 describes the mean of saturation and transmission rate parameters of Classes A-D, and the range of these parameters is reported in the parentheses. Each row represents one scenario for four classes. Four scenarios were simulated, i.e., full sized class, half sized class, full sized class with teachers vaccinated, and half sized class with teachers vaccinated.

Table S2. Mean and Range of Saturation and Transmission Rate by Class Size and Vaccination Status

Class	Class A	Class B	Class C	Class D	Overall
**Mean Saturation**					
Full sized class & not vaccinated	0.722 (0.635-0.774)	0.594 (0.514-0.727)	0.450 (0.382-0.528)	0.399 (0.294-0.463)	0.595
Half sized class & not vaccinated	0.512 (0.417-0.597)	0.452 (0.360-0.586)	0.308 (0.255-0.369)	0.290 (0.231-0.380)	0.433
Full sized class & teachers vaccinated	0.526 (0.474-0.589)	0.483 (0.402-0.588)	0.360 (0.318-0.429)	0.367 (0.323-0.394)	0.467
Half sized class & teachers vaccinated	0.397 (0.333-0.473)	0.376 (0.304-0.459)	0.269 (0.238-0.320)	0.285 (0.248-0.327)	0.357
**Mean Transmission Rate** ($\times 10^{-3}$)					
Full sized class & not vaccinated	2.653 (2.010-3.650)	1.533 (1.060-2.250)	1.492 (1.280-1.840)	0.969 (0.776-1.190)	1.856
Half sized class & not vaccinated	0.838 (0.703-1.110)	0.598 (0.430-0.809)	0.548 (0.511-0.578)	0.389 (0.306-0.458)	0.651
Full sized class & teachers vaccinated	1.748 (1.180-2.460)	1.138 (0.836-1.600)	0.935 (0.745-1.220)	0.707 (0.624-0.798)	1.273
Half sized class & teachers vaccinated	0.595 (0.449-0.752)	0.432 (0.324-0.602)	0.369 (0.323-0.419)	0.276 (0.225-0.306)	0.462

Note. Column "Overall" is the mean of saturation and transmission rate of four classes for four scenarios.

B. Probability of not observing symptomatic individuals

The emergence of symptomatic cases was significantly reduced in the half class and teacher vaccinated scenarios. We computed the probability of not observing 1st, 2nd and 3rd symptomatic individuals. Table S3 describes the mean probability that the N-th symptomatic individuals was not observed across simulations of all classes. Similar to Table S2, each row represents one scenario. Mixed effects regression models were used to predict probabilities of not observing the first, second, and third symptomatic individual from class size and vaccination, respectively (see Table S4). Both half sized classes and teacher vaccination scenarios indicate significantly higher probabilities of not observing the first three symptomatic cases than the alternative cases.

Table S3. Proportion of Simulations Without a N-th Symptomatic Individual

Without N-th symptomatic case	1st	2nd	3rd
Full sized class & not vaccinated	7.3%	29.4%	35.0%
Half sized class & not vaccinated	11.2%	48.0%	61.6%
Full sized class & teachers vaccinated	20.5%	41.0%	46.5%
Half sized class & teachers vaccinated	24.4%	58.0%	71.1%

Table S4. Mixed Effects Regression Models of Class Size and Vaccination on Not Observing Probabilities ($N = 34$)

Case Data	Parameter	Beta	S.E.	t	p
First	Half vs. Full Size	0.13	0.004	37.74	<.00001
	Not Vaccinated vs. Teacher Vaccinated	0.04	0.004	11.76	<.00001
Second	Half vs. Full Size	0.10	0.007	14.14	<.00001
	Not Vaccinated vs. Teacher Vaccinated	0.18	0.007	25.01	<.00001
Third	Half vs. Full Size	0.10	0.010	9.79	<0.00001
	Not Vaccinated vs. Teacher Vaccinated	0.26	0.010	25.56	<.00001

C. Symptomatic onset time and infections

Table S5 describes the median first three symptomatic times. Four scenarios were explored by simulation, which are full sized class, half sized class, full sized class teacher vaccinated, and half sized class teacher vaccinated. Not observed indicates the corresponding case is observed in less than half of the simulations.

Table S5. Median Time in Days to the Emergence of the N-th Symptomatic Individual.

N-th symptomatic case	1st	2nd	3rd
Full sized class & not vaccinated	3.03	8.51	12.06
Half sized class & not vaccinated	3.53	22.01	Not observed
Full sized class & teachers vaccinated	3.72	11.12	19.33
Half sized class & teachers vaccinated	4.50	Not observed	Not observed

Note. Not observed indicates that the second or third infected individual did not emerge in over half of the simulations (precluding calculation of the median). Specifically, the maximum number of symptomatic infected individuals did not exceed one or two before a stable state of 0 infected individuals emerged (all infected reverted to recovered).

4. IMPACTS OF CLASSROOM COVID-19 SIMULATION SETTINGS ON TRANSMISSIONS

A. Impact of airborne transmission

In addition to droplet transmission, airborne transmission is another potential route of COVID-19 infection. Airborne transmission occurs when individuals are infected by particles that remains airborne over interactive time. To account for airborne transmission, we generalized our modeling framework to consider the historical movements of each individual integrating both the essentially a historical spatial infection dimension (droplet transmission) function with a temporal dimension that captures residual airborne transmission. This extended model allows us to model droplet and airborne transmissions simultaneously (as described in Eq. (1)). Figure S1 shows the numerical simulations with airborne transmission for Classes A and B, with a mean airborne dissipation duration of 0.34 hour in the classroom [1] and various contributions of airborne and droplet transmissions.

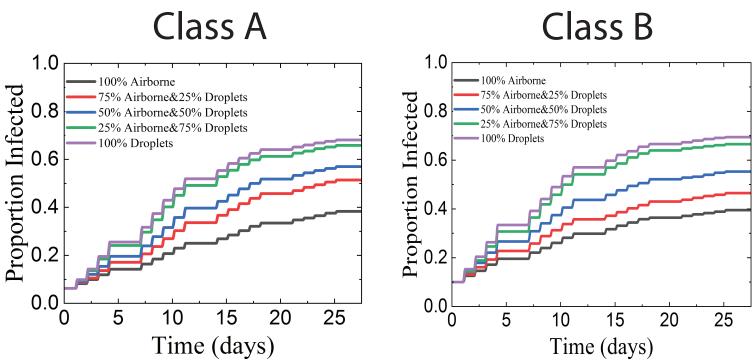

Fig. S1. Simulations of the relative influence of airborne and droplet transmission. Larger percentages of droplet transmission are associated with higher proportions of infected individuals.

B. Impacts of structured and unstructured periods of classroom time

Child and teacher behavior might vary during different class activities, where the time allotted to different activities may be under teacher/administrator control. We categorized classroom time as unstructured (free-play and transitions between activities) or as structured. Structured activities were teacher-led and primarily occurred when children were seated at tables (such as circle-time, shared book reading, meal-times, and organized play). We performed separate numerical simulations for structured and unstructured time. As illustrated in Figure S2, unstructured time appeared to be associated with a higher trajectory of infection than unstructured time, presumably because individuals were in great proximity and more mutually oriented during these periods.

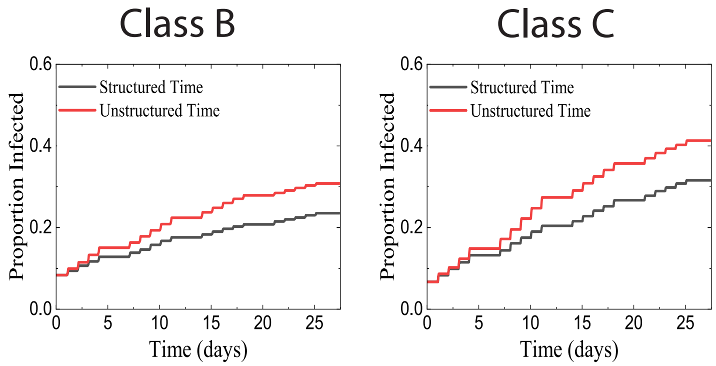

Fig. S2. Comparison of transmissions between structure and unstructured time periods. Simulation results suggest higher infection rates during unstructured time (red line) than structured time (black line).

C. Impact of σ_θ and σ_r

To understand the sensitivity of COVID-19 transmissions on the probability of infection as a function of the relative weight of distance and orientation, we employ three sets of model. Parameters ($\sigma_\theta = 45^\circ, \sigma_r = 2$m; $\sigma_\theta = 45^\circ, \sigma_r = \sqrt{2}$m; and $\sigma_\theta = 60^\circ, \sigma_r = 2$m) systematically explore different weightings of the radius between two individuals and their orientation. These scenarios are used in transmission simulations in Class A and B. As shown in Figure S3, the radius weight appears to be associated with greater changes in transmission than the orientation weighting.

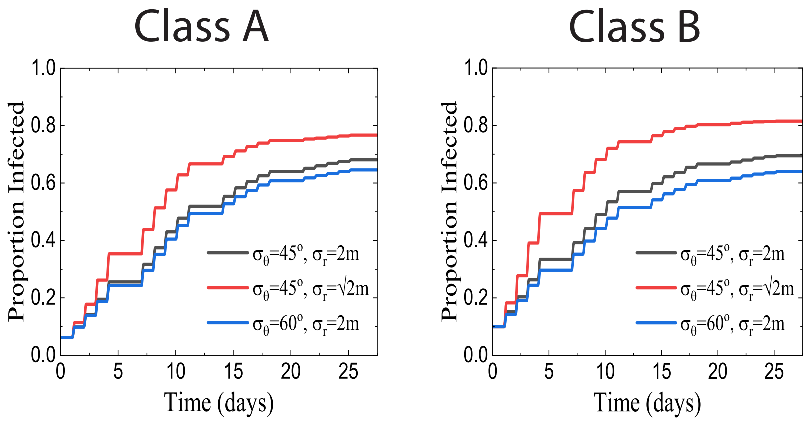

Fig. S3. Comparisons of transmissions under different σ_θ and σ_r settings in Class A and B.

D. Impact of the form of Eq. (1)

To explore how the form of the function described in Eq. (1) impacts the infection pattern, we changed the original Gaussian function to an exponential form,

$$\beta(r, \theta_1, \theta_2) = \beta_{max} \exp(-ar - b|\theta_1| - c|\theta_2|) \tag{S6}$$

where a, b, and c are three model parameters as σ_θ and σ_r in the original function. Then, we compare the simulations based on Gaussian (Eq. (1)) and exponential (Eq. (S6)) forms. Figure S4 suggests that there are no systematic differences for the Gaussian and Exponential forms.

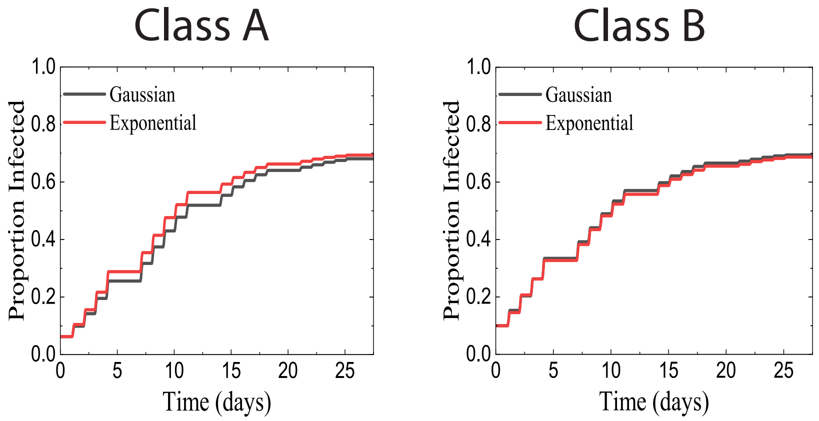

Fig. S4. Comparison of function form used to calculate infection rate differences. The black lines and red lines show the simulation results by using Gaussian and Exponential function in calculating the infection rate, respectively.

E. Impact of the duration between exposure and infectiousness

We performed numerical simulations in which the duration between exposure and infectiousness varied between 24, 48, and 72 hours. The longer the latency between exposure and infectiousness, the slower the apparent rise and peak in proportion infected (see Figure S5).

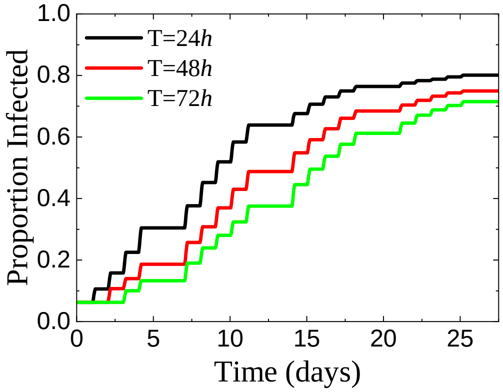

Fig. S5. Comparison of transmission simulations with different duration Ts between exposure (E) and infectiousness (I). The longest duration (green line) shows the slowest rise and peak in proportion infected whereas the shortest duration (black line) exhibits the fastest rise and peak.

F. Comparison between artificial density reduction and real low-density class observations

In the half sized class scenarios, half of children and one teacher were randomly selected to generate simulation results. Such simulations of the intervention to reduce classroom density assume no effect on the movement of the individuals who remain in the classroom. We compare the feasibility of simulated low density (half size) class scenarios with the observed low-density full sized class scenarios. Specifically, we compare observed low density classes C and D (Full Class) with the simulated half-sized classes A and B in Figure S6. While they show similar infection patterns, our simulations suggest that the artificial reductions underestimate the effect of actual reductions in density.

G. Individual heterogeneity in simulations

Our models use identical modeling parameter for all individuals. Consequently, any differences in transmission are based on observed behavioral heterogeneity, i.e., some individuals are in greater contact than others. Indeed, Figure S7 shows transmission likelihood across different individuals, in which transmission appears inhomogeneous. Figure S7 shows our simulations in Class A for different patients zero, resulting in different infection patterns. For instance, Teacher 1 being patient zero yields high infection saturation (around 0.75), whereas different children as patient zero results in different infection saturation (ranging between 0.3 and 0.8). These findings suggest that transmission variation is encoded in our models in the form of individual social interaction differences.

REFERENCES

1. R. M. Jones and L. M. Brosseau, "Aerosol transmission of infectious disease," J. occupational environmental medicine **57**, 501–508 (2015).

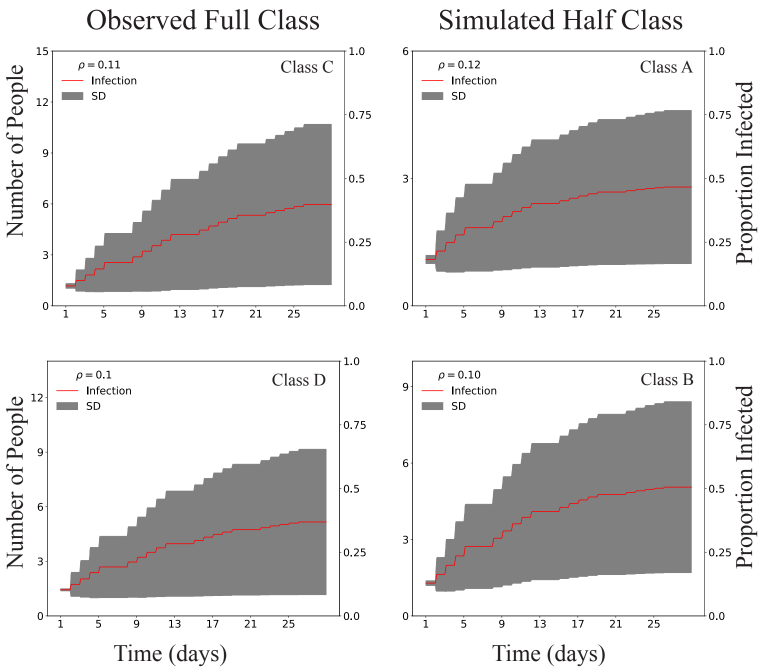

Fig. S6. Comparison between observed full class low density classrooms and simulated half class low-density classrooms. The two full class low density simulations are associated with full-sized Class C and D (left column) and the low-density simulations are associated with half sized class Class A and B (right column). Images are reproduced from Figure 3.

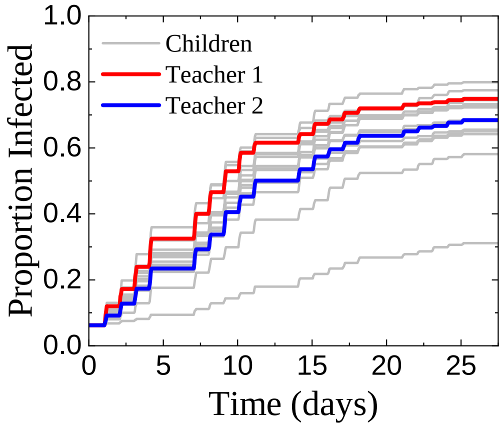

Fig. S7. Transmission heterogeneity: Different patient zero simulation results. Proportion infected over time is plotted when each child and each teacher is patient zero. Colored lines (red and blue) denote each of the teachers as patient zeros and grey lines represent each child as patient zero. It appears that the behavior of patient zeros (both teachers and children) is associated with differences in the proportion of individuals infected.
